# Development and Characterization of Biosorbent Film from Eggshell/Orange Waste Enriched with Banana Starch

**DOI:** 10.3390/polym15112414

**Published:** 2023-05-23

**Authors:** Joseph Merillyn Vonnie, Kobun Rovina, Nasir Md Nur ‘Aqilah, Xia Wen Ling Felicia

**Affiliations:** Faculty of Food Science and Nutrition, Universiti Malaysia Sabah, Kota Kinabalu 88400, Sabah, Malaysia; vonnie.merillyn@gmail.com (J.M.V.); aqilah98nash@gmail.com (N.M.N.‘A); felicialingling.97@gmail.com (X.W.L.F.)

**Keywords:** food waste, organoleptic, biosorbent, biofilm

## Abstract

The conversion of waste into a valuable product is regarded as a promising alternative to relieving the burden of solid waste management and could be beneficial to the environment and humans. This study is focused on utilizing eggshell and orange peel enriched with banana starch to fabricate biofilm via the casting technique. The developed film is further characterized by field emission scanning electron microscope (FESEM), energy dispersive X-ray spectroscopy (EDX), atomic force microscopy (AFM), X-ray diffraction (XRD), and Fourier transform infrared spectroscopy (FTIR). The physical properties of films, including thickness, density, color, porosity, moisture content, water solubility, water absorption, and water vapor permeability, were also characterized. The removal efficiency of the metal ions onto film at different contact times, pH, biosorbent dosages, and initial concentration of Cd(II) were analyzed using atomic absorption spectroscopy (AAS). The film’s surface was found to have a porous and rough structure with no cracks, which can enhance the target analytes interactions. EDX and XRD analyses confirmed that eggshell particles were made of calcium carbonate (CaCO_3_), and the appearance of the main peak at 2θ = 29.65° and 2θ = 29.49° proves the presence of calcite in eggshells. The FTIR indicated that the films contain various functional groups, such as alkane (C-H), hydroxyl (-OH), carbonyl (C=O), carbonate (CO_3_^2−^), and carboxylic acid (-COOH) that can act as biosorption materials. According to the findings, the developed film exhibits a notable enhancement in its water barrier properties, thereby leading to improved adsorption capacity. The batch experiments showed that the film obtained the maximum removal percentage at pH = 8 and 6 g of biosorbent dose. Notably, the developed film could reach sorption equilibrium within 120 min at the initial concentration of 80 mg/L and remove 99.95% of Cd(II) in the aqueous solutions. This outcome presents potential opportunities for the application of these films in the food industry as both biosorbents and packaging materials. Such utilization can significantly enhance the overall quality of food products.

## 1. Introduction

Since the 1960s, agricultural production has increased over threefold, primarily due to the expansion of cultivated land and technological advancements during the green revolution. These developments have led to higher yields, which have helped to meet the demands of a growing global population. According to the Food and Agriculture Organization (FAO) 2017 report, the world presently produces approximately 23.7 million tons of food daily [1]. However, agricultural production has significantly strained the environment on a global scale, negatively impacting soil, air, and water resources, and compromising ecosystem sustainability and human health. In addition, annual agricultural waste production is expected to reach 998 million tons. Although the global population is projected to reach nearly 10 billion by 2050, a sustainable path for agriculture has yet to be discovered [2]. Recent studies have been conducted to explore affordable and environmentally friendly biosorption materials for the remediation of pollutants. Researchers have started to focus on various agricultural waste materials, such as stone fruits, nuts and husks, peels, wood and forest residues, and plants, due to their unique chemical composition, abundant availability, renewable nature, and low cost.

Food-processing by-products of eggshells are among the most significant by-products, with eight million tons annually discarded and harming the environment. Eggshells are particularly relevant since they are abundant waste generated by the food industry. They are economical and environmentally friendly due to their availability and lack of toxic or hazardous constituents. They are as effective as activated carbon, which is more expensive. Adsorption properties of chicken eggshells include created porosity, calcium carbonate (CaCO_3_), and protein acid mucopolysaccharides [3,4]. This protein acid mucopolysaccharide has hydroxyl (-OH), carboxyl (-COOH), amino (-NH_2_), thiol (-SH), and amide (-CONH_2_) functional groups that can be employed to bind heavy metal ions [5]. Wang et al. [6] prepared a granular bentonite-eggshell composite biosorbent for removing lead (II). This granular biosorbent increased the pH of acid mine drainage from 3.00 to 6.10, indicating that the acid-neutralizing capabilities of granular bentonite-eggshell composites can minimize H+ competitive adsorption. Marković et al. [7] investigated the adsorption capacity of raw eggshells for the adsorption of copper (II) ions from aqueous samples. In another work, Annane et al. [8] utilized eggshell waste to remove cadmium (II) ions from aqueous solutions.

Orange peel, a common agricultural waste, mainly consists of cellulose, hemicellulose, pectin, and lignin components with various O/N-containing functional groups and pyranose/benzene rings [9]. It is estimated that 32 million tons of orange peel are produced annually, making it an available material for the biosorption of contaminants. Previously, the Doehlert experimental design was used to remove chromium with orange peel. Despite its low specific area, this biosorbent was proven to be an efficient material (qmax = 7.14 mg/g) [10]. Modified orange peel materials were synthesized and applied for efficient cadmium (II)-bearing wastewater purification by Tang et al. [11], showing that mercaptoacetic acid‒orange peel OH exhibits strong cadmium-sulfhydryl binding. In contrast, Altunkaynak et al. [12] examined the removal of cobalt (II) ions from aqueous solutions using orange peel waste. Pavithra et al. [13] developed a chitosan/orange peel hydrogel composite to remove heavy metal ions from wastewater containing chromium (VI) and copper (II).

The utilization of biosorbents in powder form can make it challenging to separate the phases after biosorption. To address this issue, physical modifications of these materials present a promising alternative. One possible solution is the development of biosorbent films, which can effectively remove metal ions by combining the two biomaterials mentioned above and facilitate phase separation after biosorption [14]. Native starches are considered among the most promising materials for film production, owing to their wide availability, low cost, and excellent film-forming ability [15]. In particular, green banana starch, which has a notably higher amylose concentration than potato, maize, and wheat starch and is resistant to hydrolysis, stands out among native starches [16]. This unique property plays a crucial role in the development of films, as it provides effective carrier properties for biosorbent materials. Therefore, the present study utilized banana starch as the base component for film preparation.

In this study, an eggshell/orange waste-based biosorbent film enriched with banana starch was fabricated, and its physicochemical properties were characterized. The physical and chemical attributes of the film, including thickness, density, structure, porosity, color, functional groups, and water resistance, were examined. Additionally, the film’s efficacy in removing metal ions was evaluated under various conditions, such as pH, biosorbent dosage, contact time, and initial ion concentration.

## 2. Materials and Methods

### 2.1. Materials

Raw eggshell and orange peel waste were collected from households and a nearby restaurant in Kota Kinabalu, Sabah, respectively. Green banana Saba (*Musa acuminata x balbisiana*) was purchased from fresh mart Korporasi Pembangunan Desa (KPD), Likas, Kota Kinabalu, Sabah.

### 2.2. Preparation of Eggshell and Orange Peel

The orange peel was washed and dried in an oven at 80 °C for 2 h. The dried peel was ground in a blender (Panasonic MX-337, Johor Bahru, Malaysia), sieved through a 125-micron sieve using a vibrating sieve shaker (Endecotts Minor, London, UK), and fine powder was obtained. Fresh eggshells were washed in water to remove impurities from raw materials and then dried in an oven at 50 °C for 5 h. The dried eggshells were crushed and sifted through a 125-micron sieve [17].

### 2.3. Extraction of Starch from Banana Saba (Musa acuminata x balbisiana)

The starch from unripe banana Saba pulp (*Musa acuminata x balbisiana*) was isolated using the dry extraction method, where the bananas were peeled, sliced into 2 cm slices, soaked in a 2% *w/v* citric acid solution for 5 min, and blended for 2 min. The banana pulp and the residual solution were centrifuged (Centrifuge 5430 R Eppendorf, Germany) for 5 min at 7500 rpm at room temperature, and the supernatant was discarded. The white starch sediments were dried for 18 h in a universal oven at 55 °C and sieved with aperture sizes of 125-micron [18].

### 2.4. Fabrication of Film

**Figure 1:**
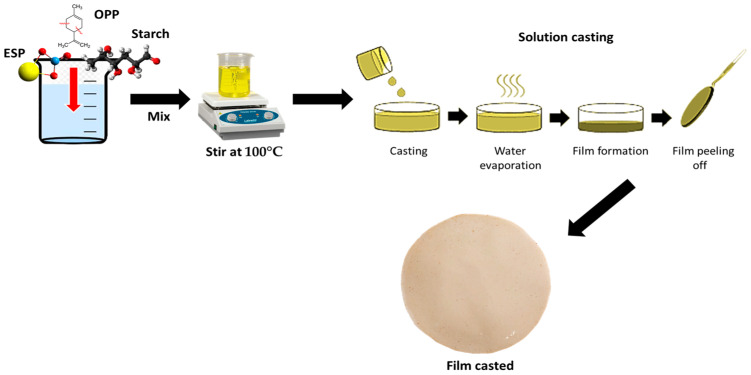
Schematic diagram illustrating the fabrication of film.

First, 1 g of eggshell powder (ESP, 0.5%) was mixed in 30 mL of distilled water and stirred for 1 h at room temperature with a magnetic stirrer to fully wet the eggshell powder particles. Following that, 6 g of banana starch flour (3%) was dissolved in 170 mL distilled water and gelatinized for 5 min at 80 °C using a hot plate (Thermo Scientific, Shanghai, China). Afterward, the eggshell powder dispersion was added dropwise to the incomplete gelatinized starch solution, and magnetic stirring was performed for 30 min. The solution was heated for 30 min at 80 °C and stirred at 400 rpm to allow complete gelatinization of the banana starch before being cooled to room temperature. After that, 1 g of orange peel powder (OPP, 0.5%) was added to the eggshell powder‒banana starch solution and thoroughly mixed. The final eggshell–orange peel powder–banana starch solutions were then poured into Petri dishes and allowed to dry for 24 h at room temperature. The development of the film is illustrated in Figure 1.

### 2.5. Method Characterization 

The FESEM images of banana starch, eggshell powder‒banana starch, orange peel powder‒banana starch, and eggshell–orange peel powder–banana starch film was obtained from field emission scanning electron microscopy equipped with energy-dispersive X-ray spectroscopy (FESEM-EDX) (JEOL, JSM-7900F, Tokyo, Japan). The film samples were coated with gold using a sputter coater (Quorum, Q150T ES PLUS, West Sussex, England) before scanning by FESEM-EDX. The surface roughness of the film was characterized by a high-resolution atomic force microscope (AFM) (Bruker MultimodeE4-Sys(8E/8J), Billerica, MA, USA) in tapping mode under ambient temperatures (22 °C). X-ray diffraction of the films was recorded by an X-ray diffractometer. The radiation used was Cu K-alpha whereas nickel metal was used as a beta filter. The range of diffraction angle was 10–80° (2θ,°). The presence of functional groups in the films was determined by the FTIR spectrometer (Agilent Cary 630, Delhi, India) in the spectrum range of 4000–400 cm^−1^.

#### 2.5.1. Thickness and Density

The thickness of banana starch, eggshell powder‒banana starch, orange peel powder‒banana starch, and eggshell–orange peel powder–banana starch films was measured at five random points using a digital micrometer (Starrett, Athol, MA, USA) with an accuracy of 0.001 mm. The average value was then calculated. Next, the original weight (W_i_) (2 cm × 2 cm) was estimated. The films were dried for 24 h at 105 °C, while the final weight (W_f_) was measured and recorded. The density was evaluated using Equation (1) below [19]:

Density = (W_i_ − W_f_)/(A × t) (1)

where W_i_ is the initial weight of the film before drying (g), W_f_ is the final weight of the film after drying (g), A is the area of the film (cm^2^), and t is the film thickness (mm).

#### 2.5.2. Color Measurement

The color of the films was determined by a colorimeter (HunterLab ColorFlex, Petaling Jaya, Malaysia) and expressed as the average L value (lightness), a value (redness/greenness), and b value (yellowness/blueness). The yellowness index and whiteness index were calculated using Equations (2) and (3). The total color difference (ΔE) was also calculated by Equation (4) [20].

Yellowness index = 142.86 (b/L)(2)

Whiteness index = 100 − (100-L)^2^+ a^2^ + b^2^ (3)

ΔE = [(L* − L)^2^ + (a* − a)^2^ + (b* − b)^2^]^1/2^ (4)

where, L*(94.60), a*(−0.42), and b*(3.24) were the standard color parameters of the calibration plate.

#### 2.5.3. Porosity Measurement

A liquid displacement method was used to calculate the percentage of film porosity. The displacement liquid was chosen to be ethanol. This method involved immersing the various dried film formulations in absolute ethanol for 24 h. After reaching equilibrium, the films were removed, and the weight of residual ethanol was recorded [21]. The porosity of the films was calculated using Equation (5) below.

Porosity (%) = [(W_2_ − W_1_ − W_3_)/(W_2_ − W_3_)] × 100 (5)

where W_1_ is the weight of the film, W_2_ is the sum of the ethanol and the immersed film, and W_3_ is the weight of residual ethanol after the removal of the film.

#### 2.5.4. Moisture Content and Water Solubility Analysis

The weight of each film (2 cm × 2 cm) was measured to obtain the initial sample weight (W_1_), and each film was dried at 105 °C until reaching a constant weight (W_2_). Moisture content was measured according to Equation (6) [22]:

Moisture content (%) = (W_1_ − W_2_)/W_1_ × 100 (6)

For the water solubility test, each film was then immersed in 50 mL of ultrapure water and kept at room temperature for 24 h. After hydration, the wet film was gently wiped using filter paper and dried in an oven at 105 °C for approximately 24 h, and the final weight (W_3_) was recorded. Therefore, the water solubility of the film (%) was determined using Equation (7) below:

Water solubility (%) = (W_2_ − W_3_)/W_2_ × 100 (7)

#### 2.5.5. Water Absorption and Water Vapor Permeability

The water absorption (or swelling) of the film was calculated as the percentage of water absorbed by the film after 1 h of immersion in water. A film sample (2 cm × 2 cm) was weighed to determine the initial weight (W_i_) before being submerged in 20 mL of water in a 50 mL container. The container was closed and left at room temperature for 1 h. After 1 h of immersion, film samples were removed, wiped with tissue paper to remove the excess water on the surface, and their wet weight (W_t_) was recorded, followed by water absorption calculation using Equation (8) below [23].

Water absorption (%) = (W_t_ − W_i_)/W_i_ × 100 (8)

The water vapor permeability values were obtained using the ASTM E96 method known as the “cup method” through a specifically developed permeability cell [24]. Each 400 mm2 film was sealed at the top of a cup with a double-sided tape containing 30 g of silica gel and laminated with aluminum foil. Each cup was then placed in a desiccator filled with water at 25 °C, and the weight was recorded every 24 h until a constant weight was obtained. The water vapor transmission rate and permeability were determined using Equations (9) and (10), respectively:

Water vapor transmission rate (WVTR) = Δm/(A × Δt)(9)

Water vapor permeability (WVP) = WVTR (x)/ΔP(10)

where Δm is the gained weight, A is the film exposed area (mm^2^), Δt is the test time (day), x is the thickness (m) of the film, and ΔP is the differential of water vapor pressure through the film (Pa). A driving force of 2339 Pa was applied as a differential vapor pressure of water.

### 2.6. Removal Efficiency Studies

The batch adsorption experiments were performed to investigate the kinetic absorption of cadmium(II) metal ions onto the eggshell–orange peel powder–banana starch film by studying the effects of the adsorbent dosage, pH, contact time, and initial concentration. The metal ions standard solution (1000 mg/L) was used to prepare 100 mg/L of metal ions stock solution. In this study, the adsorbent dosage varied from 1.2 g to 6 g. The pH was varied from 2 to 10, and it was adjusted using 0.1 M HCl and 0.1 M NaOH solution. The contact time varied from 15 to 120 min. After a predetermined time was reached, the adsorbate mixture was withdrawn and filtered. Metal ions were initially concentrated at 5 ppm to 100 ppm. Finally, the amount of metal ions left in the filtrate was determined using an Atomic Absorption Spectrophotometer (AAS). After the adsorption process, the removal percentage (%) and adsorption capacity (mg/g) were calculated using Equations (11) and (12) [13]:

Removal (%) = [(Initial metal concentration − Final metal concentration)/Initial metal concentration] × 100(11)

Adsorption capacity (qe) = [(C_0_ − C_e_) × V/m] (12)

where C_0_ and C_e_ are the initial and final metal ion concentrations (mg/L), V is the volume of the solution in liters, and m is the weight of the dry adsorbent in grams.

### 2.7. Statistical Analysis

The results will be expressed as the means ± standard deviation (SD) of 5 replicate (n = 5) measurements to ensure the accuracy of data. IBM SPSS Statistics 27 will be used to analyze the data. The physicochemical properties data were analyzed using one-way analysis of variance (ANOVA) with a post hoc Tukey test to measure significant differences (*p* < 0.05).

## 3. Results and Discussion

### 3.1. The Yield of Eggshell and Orange Peel Powder and Banana Starch

A summary of eggshell, orange peel, and banana starch yield is shown in Table 1. The drying process provided a 96.64 ± 1.94% and 21.69 ± 1.47% yield of eggshell and orange peel powder, respectively. The extraction process of banana starch provided a 13.59 ± 0.99% yield, from which approximately 1.8 kg of the banana pulp can yield 230 g of banana starch flour. According to Chávez-Salazar et al. [25], banana starch extracts yielded 5.78–12.73%.

### 3.2. Optical and Appearance of the Film

The optical, sensory evaluation, and physical appearance of banana starch, eggshell powder–banana starch, orange peel powder–banana starch, and eggshell–orange peel powder–banana starch films are shown in Table 2. The films were cast onto Petri dishes for drying. All films were uniform and had no cracks after drying. The surfaces of banana starch and orange peel powder–banana starch films were smooth with a plastic structure. However, the surfaces of eggshell powder–banana starch and eggshell–orange peel powder–banana starch films were rough due to calcium carbonate (CaCO_3_) from eggshell powder agglomerating on the starch matrix. The agglomeration leads to poor eggshell powder dispersion, which causes roughness on the film surface [26].

The color of films is a crucial factor in their overall appearance and consumer acceptance. L, a, and b values were measured to describe a three-dimensional color space. L indicates lightness and reads from 0 (black) to 100 (white). A positive a value indicates redness, while a negative a value indicates greenness; a positive b value represents yellowness, while a negative b value represents blueness [27]. The degree of total color difference from the standard color plate is measured by the color difference (ΔE) [20]. The lowest ΔE was found in the control banana starch film (9.68 ± 1.56). The eggshell powder addition generally increased the ΔE value in the eggshell–orange peel powder–banana starch (39.22 ± 0.86) and eggshell powder–banana starch (24.65 ± 1.45) films. Chareonsuk et al. [28] found that eggshell powder can induce overall color change in biocomposite films.

Considering the a value, it was observed that the a values showed no significant difference (*p* > 0.05) between banana starch (−5.68 ± 0.30) and orange peel powder–banana starch (−5.52 ± 0.44) film. However, the addition of eggshell powder yielded a slightly higher a value in eggshell powder–banana starch (−3.86 ± 0.15) and eggshell–orange peel powder–banana starch (−3.86 ± 0.11) films compared with the control banana starch film. The L and b values were significantly different when compared between the films (*p* < 0.05). The control banana starch film (93.32 ± 1.53) had the highest L value, followed by orange peel powder–banana starch (84.90 ± 2.75), eggshell powder–banana starch (72.50 ± 1.70), and eggshell–orange peel powder–banana starch (61.06 ± 1.17) films. It was observed that the addition of eggshell powder made the eggshell–orange peel powder–banana starch film darker and had the lowest L value compared with the control banana starch film. In addition, the eggshell–orange peel powder–banana starch film (54.47 ± 0.86) also had the lowest whiteness index value compared with other films. Incorporating polymer with orange peel contributed to a yellower and darker brown composite film.

On the contrary, the control banana starch film (11.14 ± 1.54) had the lowest b value, while the orange peel powder addition increased the b values of the orange peel powder–banana starch (19.66 ± 0.15) and eggshell–orange peel powder–banana starch (23.26 ± 0.33) films. The b value indicated that the yellow color intensity of orange peel powder–loaded films increased when compared with control banana starch and eggshell powder–banana starch films. In addition to that, the yellowness index values of the films changed significantly (*p* < 0.05) with the addition of orange peel powder. The results showed that eggshell–orange peel powder–banana starch (54.43 ± 0.57) and orange peel powder–banana starch (33.11 ± 1.18) films had the highest yellowness index values compared with control banana starch (17.09 ± 2.61) and eggshell powder–banana starch (26.68 ± 1.69) films. These study results were similar to other studies that observed that films containing orange peel powder had a yellowish color owing to the existence of carotenoid pigments in the orange peel [17,29]. Hence, adding orange peel powder increased the greenness and yellowness values of the films due to the orange peel powder having relatively low a and high b values [30].

### 3.3. Morphology Analysis

**Figure 2:**
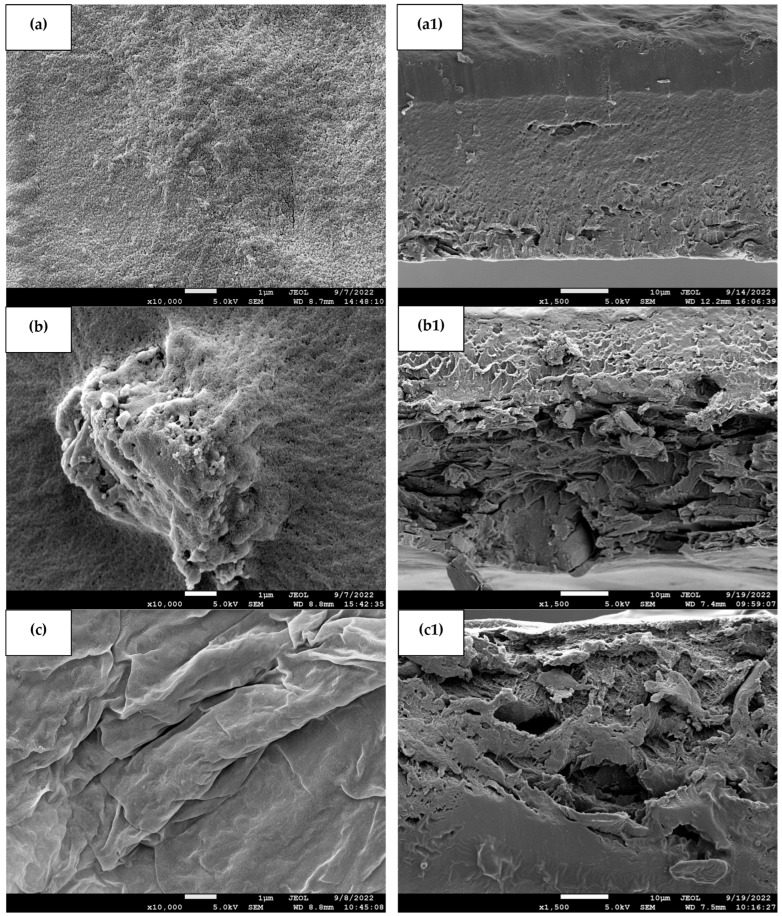

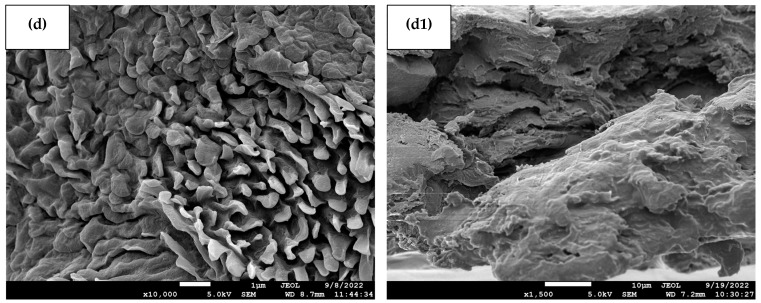
FESEM images of (**a**) BS, (**b**) ESP-BS, (**c**) OPP-BS, and (**d**) ESP-OPP-BS films at a magnification of ×10,000 with the cross-section images of (**a1**) BS, (**b1**) ESP-BS, (**c1**) OPP-BS, and (**d1**) ESP-OPP-BS films at a magnification of ×1500.

Figure 2 exhibits the surface and cross-section FESEM images of the films at ×10,000 and ×1500, respectively. The surface of the banana starch film (Figure 2a) was smooth and homogenous without pores and cracks compared with other films. Similar results were obtained by Vonnie et al. [16] and Tibolla et al. [31]. This may be related to their high content of starch, especially amylose, and low content of other macromolecules. The presence of starch molecules formed stable complexes in the polymer matrix, resulting in a homogeneous polymeric structure of the banana starch film [32]. Based on Figure 2b, the addition of eggshell powder to the starch matrix showed that the surface of the eggshell powder–banana starch film was porous and irregularly shaped. As a result of the addition of eggshell powder to banana starch, agglomeration was formed in the polymeric film matrix. A nonuniform dispersion of eggshell powder particles in the banana starch film matrix decreased the interaction between eggshell powder particles and the banana starch film matrix [33]. On the other hand, Figure 2c reveals that the surface of the orange peel powder–banana starch film was rough and nonuniform without any cracks, which enhanced the target analytes’ interaction [34]. However, Figure 2d shows a porous surface of the eggshell–orange peel powder–banana starch film without cracks. The addition of orange peel powder improved the structure of eggshell powder particles with no agglomeration occurring. Yaradoddi et al. [35] found that orange peel waste is largely composed of cellulose, pectin, lignin, and hemicellulose. Due to the high content of these components, the eggshell powder particles dispersed and adhered well to the polymer matrix.

In terms of cross-section, the banana starch film (Figure 2(a1)) exhibited a continuous and compact matrix. Meanwhile, Figure 2(b1) revealed that eggshell powder–banana starch film has a microporous structure according to the reported literature [36]. Its porous external and internal layers make raw eggshell an appealing bio-adsorbent due to its high adsorption capacity. The external surface of the eggshell consists mainly of calcium carbonate (CaCO_3_). In contrast, the inner layer consists primarily of uncalcified fibrous membranes composed mainly of organic compounds [37]. In Figure 2(c1), the cross-sectional image of an orange peel powder–banana starch film also shows a slightly porous structure. A similar study by Pavithra et al. [13] found that composite films made of orange peel powder had pore-like and curved structures. Due to the presence of these pores, the orange peel powder will have a better capacity for target analytes adsorption. This is because they can diffuse into and get trapped at the active sites. By combining eggshell powder and orange peel powder, the film becomes highly porous and has more adsorption sites for analytes, as shown in Figure 2(d1). As the eggshell–orange peel powder–banana starch films exhibit porous surface textures, they enhance the surface area of the adsorbent, allowing adsorption to occur while also providing a nonadhesive appearance that prevents agglomeration. Biosorption of analytes from an aqueous medium will be facilitated by improving porosity and fracture structure. In view of this, the eggshell–orange peel powder–banana starch film has been declared suitable for the removal of various target analytes.

**Figure 3:**
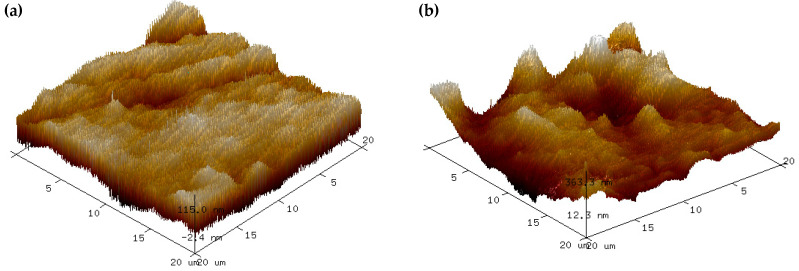

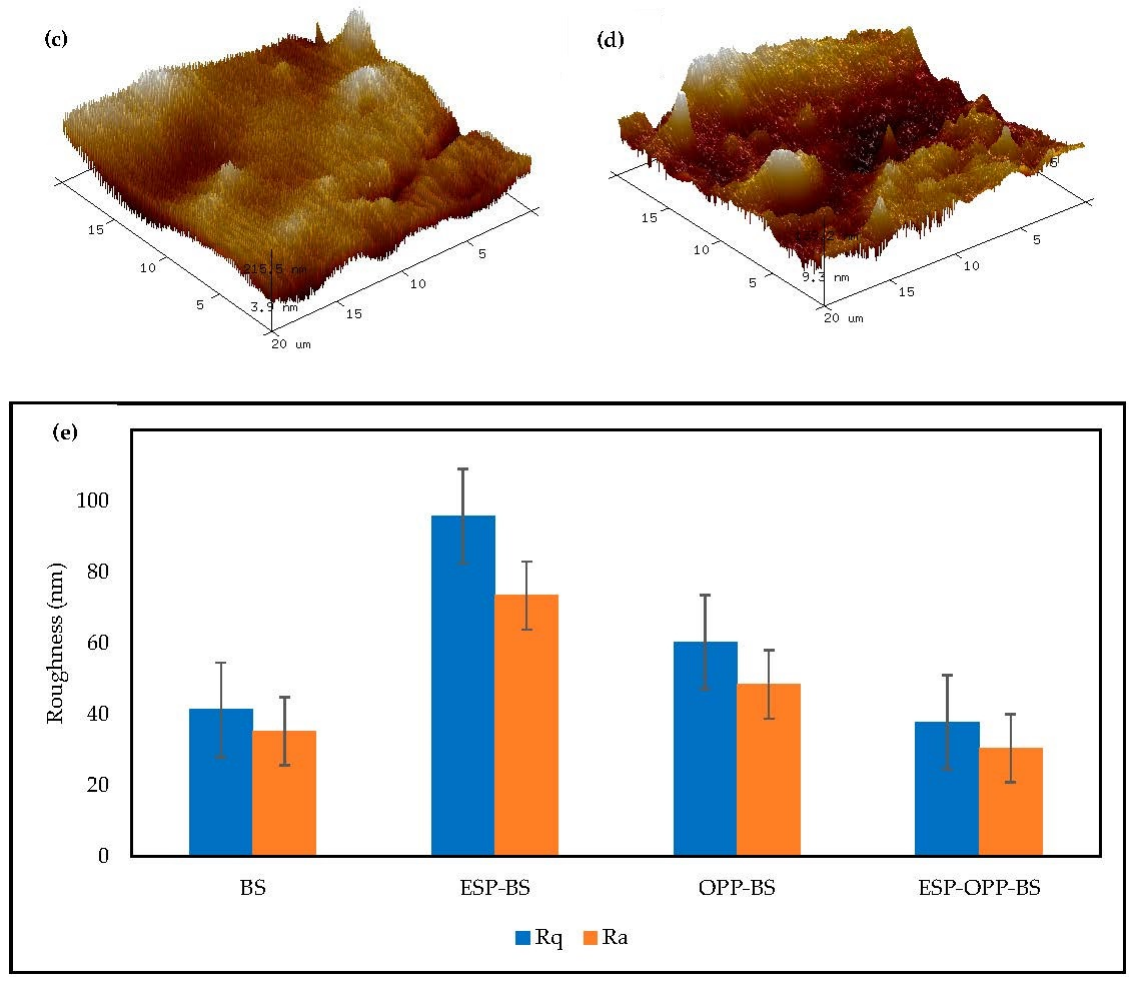
AFM three-dimensional images of the (**a**) BS, (**b**) ESP-BS, (**c**) OPP-BS, and (**d**) ESP-OPP-BS films, and (**e**) the root-mean-square roughness (Rq) and average roughness (Ra) on the surface of films.

An AFM was used to observe the surface structure in deeper insight. The AFM three-dimensional images with the root-mean-square roughness (Rq) and average roughness (Ra) values in Figure 3 clearly demonstrate that the surface of the eggshell powder–banana starch film (Figure 3b) and orange peel powder–banana starch film (Figure 3c) was rougher than the surface of the banana starch film (Figure 3a) and of the eggshell–orange peel powder–banana starch film (Figure 3d), which agreed well with FESEM observation. The eggshell powder–banana starch and orange peel powder–banana starch film obtained 73.3 and 48.3 nm of Ra values, respectively. In contrast, the banana starch and eggshell–orange peel powder–banana starch film had the lowest Ra values with 35.2 and 30.4 nm, respectively (Figure 3e). As a result of the incorporation of eggshell powder and orange peel powder together with starch, the film has been able to maintain its porous structure while improving its surface roughness with the absence of agglomeration.

**Figure 4:**
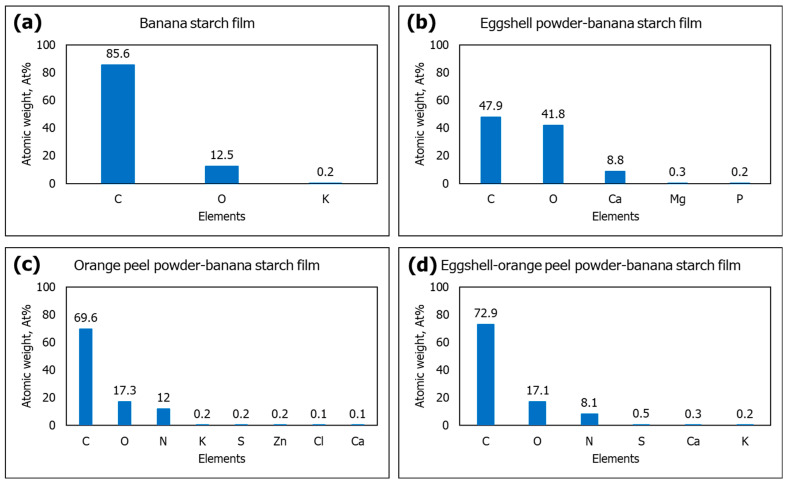
EDX atomic weight (at%) of (**a**) BS, (**b**) ESP-BS, (**c**) OPP-BS, and (**d**) ESP-OPP-BS films.

The elemental composition analysis of the films was determined using EDX. The major elements found in the films are carbon (C) and oxygen (O). The EDX results indicate that 85.6% carbon, 12.5% oxygen, and 0.2% potassium were detected in the banana starch film (Figure 4a). According to Pongsuwan et al. [38], banana powder contains mostly carbon (53.9%) and oxygen (42.5%), which indicates it is a high source of organic matter. The EDX of the eggshell powder–banana starch film (Figure 4b) demonstrates that the eggshell powder particles contain calcium (8.8%), oxygen (41.8%), magnesium (0.3%), and phosphorus (0.2%), with the presence of carbon (47.9%). The results of these analyses are comparable to Kim et al. [39], who found that chicken eggshell powder primarily consisted of calcium, oxygen, and carbon, along with minor amounts of magnesium. These showed that the eggshell powder particles were made up of CaCO_3_ and contained a significant amount of carbon in the graphite.

The orange peel powder–banana starch film (Figure 4c) contains carbon (69.6%), oxygen (17.3%), and nitrogen (12.0%), accompanied by small amounts of potassium (0.2%), sulfur (0.2%), zinc (0.2%), chlorine (0.1%), and calcium (0.1%). The percentages of carbon and oxygen elements in the eggshell–orange peel powder–banana starch film are 72.9% and 17.1%, respectively (Figure 4d). This is due to extensive hydrogen bonding interactions between eggshell powder, orange peel powder, and starch. Eggshell powder and orange peel powder hydroxyl groups can bind with starch hydroxyl groups, reducing the number of polymer chain molecules and improving film stability [40]. Nitrogen (8.1%), sulfur (0.5%), calcium (0.3%), and potassium (0.2%) were also present in the eggshell–orange peel powder–banana starch film. Due to the abundance of oxygen surface atoms present in orange peel powder, more biosorption activity could be achieved by interacting with the target analytes [41]. The mineral components, such as magnesium, phosphorus, potassium, nitrogen, sulfur, zinc, and chlorine were nutrients that the banana and orange plants accumulated during growth [42].

**Figure 5:**
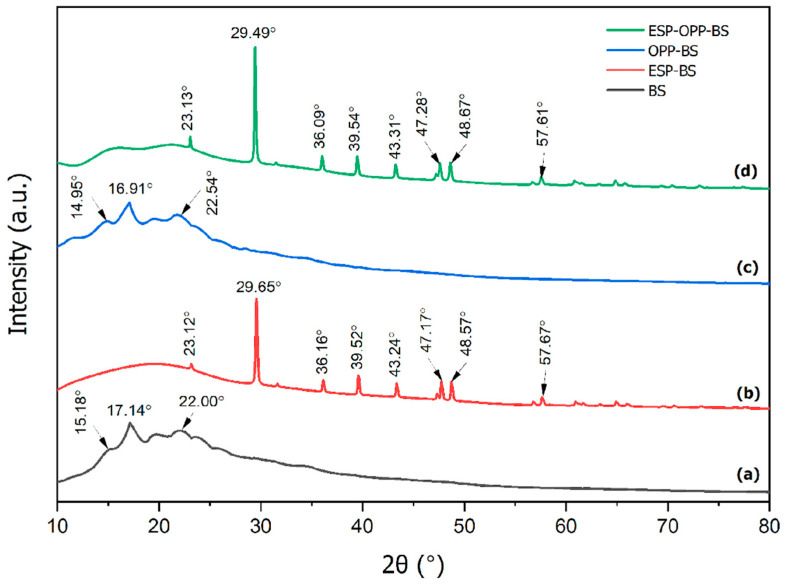
XRD spectrum of (**a**) BS, (**b**) ESP-BS, (**c**) OPP-BS, and (**d**) ESP-OPP-BS films.

The amorphous and crystalline states of films composed of banana starch, eggshell, and orange peel powders were investigated by XRD analysis. For the banana starch film, starch particles have a semi-crystalline nature, as shown in Figure 5a. As shown in XRD patterns, banana starch granules displayed points of a small peak at 15.18°, a strong peak at 17.14°, and a broad peak at 22.00°. Generally, starch granules originating from different sources exhibit varying crystallization characteristics. As reported previously, banana starch displays B-type crystallinity regardless of the variety and starch source [43]. For orange peel powder–banana starch film (Figure 5c), the XRD pattern showed a small peak at 14.95°, a strong peak at 16.91°, and a broad peak at 22.54°.

Similar XRD peaks for orange peel powder were found by Akinhanmi et al. [44] at 2θ = 16.3° and 22.3° for cellulose, which indicates it is an amorphous cellulose structure. Figure 5b,d illustrate the amorphous structure of banana starch and orange peel powder with the crystallinity of the eggshell powder particles, which contain a main peak at 2θ = 29.65° and 2θ = 29.49°, as well as smaller peaks at 2θ = 23.12°, 36.16°, 39.52°, 43.24°, 47.17°, 48.57°, 57.67°, and 2θ = 23.13°, 36.09°, 39.54°, 43.31°, 47.28°, 48.67°, 57.61°, which confirm the presence of calcite in eggshells [8]. Calcite is the most stable form of CaCO_3_ at room temperature, and it is found in eggshells and other rigid materials [45]. The XRD results of both films show that the composition of the uncalcined eggshell is mainly made up of calcium-magnesium carbonate (CaCO_3_–MgCO_3_), with the absence of a calcium oxide (CaO) peak [46]. This confirms the composition of the eggshell, which is mostly made up of CaCO_3_.

### 3.4. Infrared Spectroscopy

**Figure 6:**
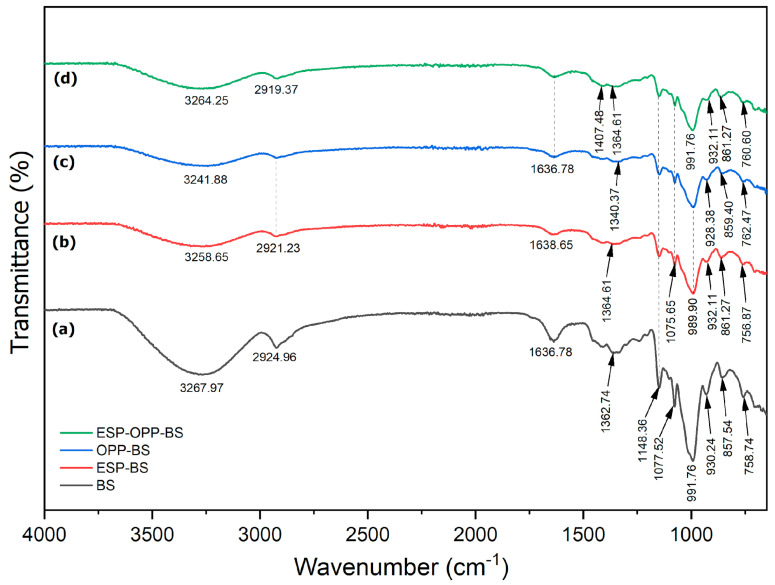
FTIR spectra of (**a**) BS, (**b**) ESP−BS, (**c**) OPP−BS, and (**d**) ESP−OPP−BS films.

Figure 6 shows a spectrum of the FTIR-spectroscopic analysis performed on the films to confirm the existence of diverse functional groups. The film’s spectra exhibited nearly identical peaks due to their chemical similarities in starch and cellulose. The films demonstrated that peak bands at 3241.88 to 3267.97 cm^−1^ were identified as complex vibrational -OH stretching of hydrogen-bonded hydroxyl group [47]. Bands at 2919.37 to 2924.96 cm^−1^ were typical of -CH bond stretching of the alkanes group [48]. The peak bands at 1636.78 to 1638.65 cm^−1^ were the effects of vibrational C=O stretching of carbonyl groups of amide I resulting from water adsorbed in amorphous amylose regions, while bands at 1362.74 cm^−1^ and 1364.61 cm^−1^ were in the region of amide III. Bands in the amide III region were caused by C-N bond stretching and N-H bond inflection. Those bands indicate residual proteins in the starch film [48]. Furthermore, the prominent bands detected at 1148.36 cm^−1^ are caused by starch stretching of the C-C and C-O groups. The peaks at 1077.52 cm^−1^ and 1075.65 cm^−1^ were attributed to the C-O bond stretching of carboxylic acid (-COOH) groups, which has a major influence on the adsorption process and the effectiveness of adsorbing target analytes through ion exchange. The bending of COH and -CH groups caused by the cellulose component resulted in the peak at 1340.37 cm^−1^ of the orange peel powder–banana starch film (Figure 6c) [49]. A peak of eggshell powder particles was found in the eggshell–orange peel powder–banana starch film at 1407.48 cm^−1^ (Figure 6d), which is closely related to the presence of carbonate minerals inside the film matrix. The carbonate absorption peak at 1407.48 cm^−1^ is also notable and supports the results of Onwubu et al. [50]. They mentioned that carbonate-based materials often detected the C=O bond’s broad stretching frequency in carbonate ions. The starch C-O bond’s vibrational stretching was found from 989.90 to 991.76 cm^−1^. Peak bands between 928.38 and 932.11 cm^−1^ were caused by the saccharide structure. The bands are attributed to C-O, C-O-H, and C-O-C bonds stretching in the glycosidic backbone of starch. According to Alimi and Workneh [51], changes in the position and intensity of the bands may be due to the existence of ɑ-1,6 glycosidic linkages of amylopectin. Furthermore, the peak band found at 861.27 cm^−1^ in eggshell powder–banana starch (Figure 6b) and eggshell–orange peel powder–banana starch films was related to asymmetric stretching of the C-H bond assigned, as well as in-plane and out-of-plane deformation, confirming the presence of CaCO_3_. With the addition of eggshell powder, the peak at 3267.97 cm^−1^ of -OH stretching and the peak at 2924.96 cm^−1^ of -CH stretching were reduced to 3258.65 cm^−1^ and 2919.37 cm^−1^, respectively. These findings revealed an increase in the number of hydrogen bonds formed between the starch molecules and the eggshell powder particles, which resulted in a decrease in the vibrational frequency of the pyranoid ring skeleton and a shift in the absorption bands to a lower wavenumber. The addition of eggshell powder enhanced hydrogen bonding, decreasing water adsorption sites and strengthening the compact structure of composite films by reducing free spaces in the film network. Thus, the eggshell powder–banana starch and eggshell–orange peel powder–banana starch films have consequently improved their resistance to water and oxygen.

### 3.5. Thickness, Density, Porosity, and Water Barrier Property

The thickness, density, porosity, and water barrier property values of the films are shown in Table 3. Film thickness is a crucial factor in determining the physical properties of a film. The results showed that the thickness of all the films changed significantly (*p* < 0.05) from 0.02 ± 0.01 to 0.07 ± 0.01 mm. The eggshell–orange peel powder–banana starch film presented the highest thickness value (0.07 ± 0.01 mm). The thickness of the control banana starch film was 0.02 ± 0.01, whereas the thickness of the eggshell powder–banana starch film was increased to 0.05 ± 0.01 mm due to the presence of CaCO_3_ particles in the eggshell powder, which improved the intermolecular bonding between ESP and starch film matrix [52]. It was also found that the addition of orange peel powder increased the orange peel powder–banana starch film thickness to 0.04 ± 0.01 mm. This was similar to Terzioğlu et al. [30] findings, which resulted in an increase in film thickness due to the structure of the incorporated orange peel powder. This is because the powder was made up of both soluble and insoluble fibers. Moreover, the increase in thickness associated with orange peel loading was due to an increase in the size and solid content of the orange peel agglomerates [53]. Thus, the addition of eggshell and orange peel powder can significantly increase the thickness of starch films.

At the same time, the density of all the films ranged from 0.09 ± 0.01 to 0.26 ± 0.14, and it was observed that they increased (*p* < 0.05) with the addition of eggshell and orange peel powders. No significant difference (*p* > 0.05) was observed between the density of eggshell powder–banana starch (0.13 ± 0.03) and orange peel powder–banana starch (0.20 ± 0.08) films. However, the control banana starch film (0.26 ± 0.14) had the highest density, which might be due to a more compact structure of the starch film, which can be seen in the cross-section image (Figure 2a1). Meanwhile, the eggshell–orange peel powder–banana starch film (0.09 ± 0.01) had the lowest density, which may be due to the porous structure of the film. The existence of pores can result in a lower density in composite film [54].

It is essential to determine the porosity percentage of the films in order to measure properties, such as water absorption and water vapor permeability. Furthermore, an ideal biosorbent film requires porosity for effective adsorption. Here, the porosity percentage of the films was evaluated, and the results are shown in Table 3. The results show that the porosity of the banana starch film significantly increased (*p* < 0.05) from 36.52 ± 1.82% to 82.53 ± 3.94% with the addition of eggshell powder and orange peel powder. This is due to the eggshell powder containing high CaCO_3_ concentrations, high porosity, and availability of functional groups [55], which is confirmed by the FTIR results. According to Kim et al. [39] and Dey et al. [41], chicken eggshell powder and spent orange peel had mesopore structures with 144.649 Å and 56.4 Å of pore size, respectively. Thus, the results obtained clearly indicate that both eggshell powder and orange peel powder are effective as bio-adsorbent materials for target analytes removal.

Moisture content, water solubility, water absorption, and water vapor permeability of films were measured to determine the film’s water barrier properties. Moisture content is the quantity of moisture present or absorbed by the film from the environment and released as vapor at high temperatures (80–100 °C) [56]. It is expressed as a percentage of the total mass of the material [57]. The amount of water molecules available in the network microstructure of the film contributes to the moisture content of composite films. Referring to Table 3, the moisture content of the banana starch film was found to be the highest at 11.23 ± 0.72% followed by orange peel powder–banana starch film with 10.59 ± 0.41%. As a result of starch’s hydrophilic nature and its hydroxyl groups (-OH), water molecules or moisture are absorbed by it from the environment [58]. The moisture content in the eggshell powder–banana starch and eggshell–orange peel powder–banana starch film was significantly decreased (*p* < 0.05) to 9.98 ± 0.46 and 10.23 ± 0.40, respectively compared with control banana starch film because of the discontinuity of the starch matrix, thus making space for water to escape from the structure of the starch [59]. The interaction of the eggshell powder and orange peel powder with the polymer matrix resulted in the formation of hydrogen bonds with hydroxyl groups (-OH). There was less starch–starch interaction due to the biosorbent matrix, which resulted in a reduction in the water absorption capacity of the film [60].

The water solubility of the film is an extremely critical and important parameter, especially if the film is directly exposed to water [61]. The control banana starch film (20.20 ± 2.29%) displayed the highest solubility in water followed by orange peel powder–banana starch film (16.66 ± 1.64%). The values for solubility in water were seen to decrease significantly (*p* < 0.05) upon adding eggshell powder in both eggshell powder–banana starch (13.06 ± 1.31%) and eggshell–orange peel powder–banana starch (13.58 ± 1.01) films. In this study, the water solubility of banana starch and the composite films followed the same pattern as the moisture content. This showed that eggshell powder reduced the hydrophilicity of films. In this case, the hydroxyl groups (-OH) of degraded starch interacted with the functional groups of ES molecules, resulting in a cross-linking network between them. Furthermore, eggshell powder–banana starch and eggshell–orange peel powder–banana starch films were less attractive to water molecules because of the reduced number of free -OH groups, which also slowed down the release of free polymer chains from starch to water, and caused eggshell powder–banana starch and eggshell–orange peel powder–banana starch films to be less soluble in water than banana starch films. A slight decrease in orange peel powder–banana starch film was due to essential oil compounds in the orange peel powder particles that were insoluble in water. Moreover, this decrease was also caused by cellulose insolubility in orange peel powder [62].

As banana starch has free hydroxyl groups (-OH), it can exhibit high water adsorption, swelling, and solubility, resulting in one of the major disadvantages of banana starch composite films. The percentage of water adsorption of the films can be found in Table 3. As can be seen from the results, all films adsorbed water very well during the 60 min immersion process to reach the saturation level. Even though starch is one of the most hydrophilic materials, adding the eggshell powder to the material can obviously enhance its water resistance properties. The water adsorption value for the control banana starch film was 164.07 ± 47.30%, while it was reduced significantly (*p* < 0.05) to 88.64 ± 8.87% and 93.88 ± 28.48% for the eggshell powder–banana starch film and eggshell–orange peel powder–banana starch film, respectively. Water adsorption decreased significantly with eggshell powder loading due to the hydrophobic nature of eggshell powder particles as well as their interactions with the banana starch film matrix (decrease in the number of free hydroxyl groups) [23].

Film water vapor permeability refers to the impedance of the material to water vapor from the environment. Relatively speaking, the banana starch film showed a 2.20 ± 0.41 gs^−1^ m Pa (×10^−12^) of water vapor permeability, which was increased to 6.47 ± 2.59 gs^−1^ m Pa (×10^−12^) in the eggshell–orange peel powder–banana starch film. The hydrophobic nature of eggshell powder resulted in a significant reduction in water adsorption due to the reduced amount of free OH groups that can form tortuous film structures, which increased the effective diffusion path length of water. On the other hand, adding eggshell powder particles could create channels or microvoids at the interface of the matrix, increasing water vapor permeability and providing large direct paths for water molecules [63]. This result was in good accordance with the FESEM observation in Figure 2 and FTIR analysis in Figure 6, in which the wide pores of eggshell and orange peel composites caused the water vapor permeability of the eggshell–orange peel powder–banana starch film to be the highest.

### 3.6. Effects on pH, Biosorbent Dosage, Contact Time and Initial Concentration

**Figure 7:**
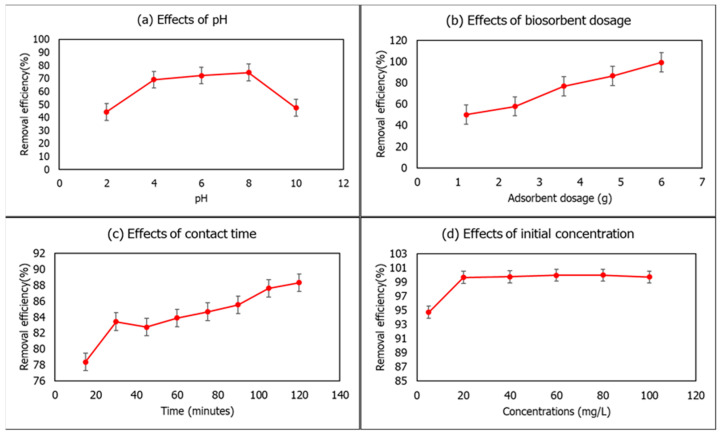
Effects of (**a**) pH, (**b**) biosorbent dosages, (**c**) contact time, and (**d**) initial concentration on the removal efficiency of Cd(II) ions by ESP-OPP-BS film.

The pH of a solution is a fundamental parameter that determines its removal efficiency (%) due to the fact that the pH of a solution affects the active sites on the biosorbent. Initially, the pH of the aqueous solution was 2.3. Here, the pH of the 5 mg/L of Cd(II) ion solution varied from 2 to 10 at a constant 1 g of biosorbent dose and 60 min contact time to evaluate the optimum pH for the removal of Cd(II) at room temperature. As the pH increased, the removal efficiency (%) increased and the maximum value was obtained at pH 8, which, then decreased drastically at pH 10 (Figure 7a). Similar findings were reported by Bhutto et al. [64]. After the optimum pH was achieved, the percentage removal of Cd(II) decreased as its pH increased from 8 to 9 for Cd(II) ions. Therefore, pH 8 was selected for further biosorption experiments of Cd(II) ions. It was also observed that the biosorption of Cd(II) ions from an aqueous solution increased with an increase in the amount of eggshell–orange peel powder–banana starch film ranging from 1.2 to 6.0 g at pH 8, Cd(II) ions concentration of 5 mg/L, and 60 min of contact time at room temperature. The highest quantitative adsorption of Cd(II) ions was observed at 6.0 g of the eggshell–orange peel powder–banana starch film (Figure 7b). As for the 6.0 g biosorbent dosage, the removal rates of Cd(II) ions reach 99.30%. An increased eggshell–orange peel powder–banana starch film dose suggested increased adsorption sites and surface area accessible for adsorption. Thus, 6.0 g of the eggshell–orange peel powder–banana starch film dosage was selected for Cd(II) further study.

Next, the eggshell–orange peel powder–banana starch film has been examined for Cd(II) ions removal with contact times ranging from 15 to 120 min at fixed intervals of 15 min. The optimum contact time for the removal of Cd(II) ions was observed at 120 min (Figure 7c). The biosorbent surface has many active sites available to bind the metal ions at the starting process, thus allowing the removal of metal ions to occur quickly [64]. The percentage removal of metal ions increased from 78.39% to 88.32% for Cd(II) ions. The effect of the initial concentration of Cd(II) solutions was analyzed for 5, 20, 40, 60, 80, and 100 mg/L concentrations by maintaining other factors, such as pH 8, 6.0 g of biosorbent dosage, 120 min of contact time, and temperature as constant room temperature. Upon increasing the metal ion concentration from 5 to 80 mg/L, the percentage elimination of Cd(II) increased from 94.72 to 99.95% (Figure 7d), and then, decreasing at 100 mg/L concentrations. The removal percentage decreases when the Cd(II) ions concentration increases to 100 mg/L due to the lower value of the surface area to the metal ions concentration ratio and the decrease in the availability of metal-ion binding sites [13]. The overall results revealed that the eggshell–orange peel powder–banana starch film could remove Cd (II) ions with 99.95% of removal rates at 100 mg/L concentrations. This study is also compared with other findings shown in Table 4.

## 4. Conclusions

In this research, the eggshell–orange peel powder–banana starch film was prepared by a solution-casting method. According to FESEM analysis, the eggshell powder particles dispersed and adhered well to the starch matrix. In addition, the incorporation of orange peel powder improved the structure of eggshell powder particles with no agglomeration occurring, and eggshell–orange peel powder–banana starch films exhibited a porous surface with no cracks. Based on the AFM analysis, the XRD analysis revealed that the film has a semi-crystalline structure containing the main peaks at 2θ = 29.65° and 2θ = 29.49° due to the presence of calcite from eggshell particles. Furthermore, FTIR analysis showed that the eggshell–orange peel powder–banana starch film has many functional groups that contributed to the formation of hydrogen bonds and the compactness of composite films, as well as its potential to produce a strong absorption band that is strongly correlated with carbonate minerals in composite films. By integrating eggshell and orange peel powder, the eggshell–orange peel powder–banana starch film exhibits improved thickness, density, porosity, moisture content, water solubility, absorption, and vapor permeability when compared with banana starch films. Eggshell and orange peel powder can improve the mechanical strength, barrier properties, and hardness of the films without using cross-linking agents. In addition to that, the removal efficiency experiments conducted obtained the optimum pH at pH 8, 6 g of biosorbent dosage, 120 min of contact time, and 80 mg/L of initial concentration of Cd(II) ions. The biosorbent film successfully removed 99.69 mg/L of Cd(II) ions. Hence, this eggshell–orange peel powder–banana starch film is suitable to be used for the adsorption of target analytes, such as toxins or dyes that are present in food products.

## Figures and Tables

**Table 1:** The yield of eggshell and orange peel powder and banana starch.

Powder	Yield (%)	Photo
Eggshell	96.64 ± 1.94	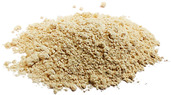
Orange peel	21.69 ± 1.47	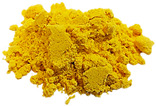
Banana starch	13.59 ± 0.99	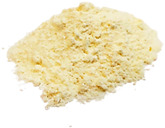

**Table 2:** The optical and physical appearance of the films.

Film	BS	ESP-BS	OPP-BS	ESP-OPP-BS
L	93.32 ± 1.53 ^d^	72.50 ± 1.70 ^b^	84.90 ± 2.75 ^c^	61.06 ± 1.17 ^a^
a	−5.68 ± 0.30 ^a^	3.86 ± 0.15 ^b^	−5.52 ± 0.44 ^a^	−3.86 ± 0.11 ^b^
b	11.14 ± 1.54 ^a^	13.54 ± 0.91 ^b^	19.66 ± 0.15 ^c^	23.26 ± 0.33 ^d^
ΔE	9.68 ± 1.56 ^a^	24.65 ± 1.45 ^c^	19.86 ± 1.43 ^b^	39.22 ± 0.86 ^d^
Yellowness index	17.09 ± 2.61 ^a^	26.68 ± 1.69 ^b^	33.11 ± 1.18 ^c^	54.43 ± 0.57 ^d^
Whiteness index	85.80 ± 1.98 ^d^	69.08 ± 1.43 ^b^	74.53 ± 1.71 ^c^	54.47 ± 0.86 ^a^
Color	White	Light brown	Light yellow	Yellow-brown
Texture	Dry, smooth	Dry, rough, brittle	Dry, smooth	Dry, hard, rough
Smell	Odorless	Sulfur smell	Orange fragrance	Soft orange fragrance
Appearance	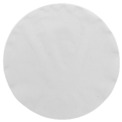	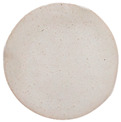	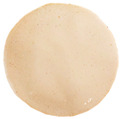	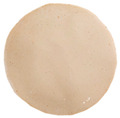

Different superscript lowercase letters within the same row indicate significant differences between film samples by Tukey’s test (*p* < 0.05) with data mean (n = 5). BS: Banana starch; ESP-BS: Eggshell powder–banana starch; OPP-BS: Orange peel powder–banana starch; ESP-OPP-BS: Eggshell–orange peel powder–banana starch.

**Table 3:** The physical parameters values of the films.

Parameters	BS	ESP-BS	OPP-BS	ESP-OPP-BS
Thickness (mm)	0.02 ± 0.01 ^a^	0.05 ± 0.01 ^b^	0.04 ± 0.01 ^b^	0.07 ± 0.01 ^c^
Density (g/cm^3^)	0.26 ± 0.14 ^b^	0.13 ± 0.03 ^ab^	0.20 ± 0.08 ^ab^	0.09 ± 0.01 ^a^
Porosity (%)	36.52 ± 1.82 ^a^	78.51 ± 3.24 ^bc^	76.57 ± 2.92 ^b^	82.53 ± 3.94 ^c^
Moisture content (%)	11.23 ± 0.72 ^a^	9.98 ± 0.46 ^bc^	10.59 ± 0.41 ^ab^	10.23 ± 0.40 ^bc^
Water solubility (%)	20.10 ± 2.29 ^c^	13.06 ± 1.31 ^a^	16.66 ± 1.64 ^b^	13.58 ± 1.01 ^a^
Water adsorption (%)	164.07 ± 47.30 ^b^	88.64 ± 8.87 ^a^	123.29 ± 28.83 ^ab^	93.88 ± 28.48 ^a^
Water vapor permeability (gs^−1^ m Pa) (×10^−12^)	2.20 ± 0.41 ^a^	5.22 ± 2.08 ^a^	3.95 ± 2.29 ^a^	6.47 ± 2.59 ^a^

Different superscript lowercase letters within the same row indicate significant differences between film samples by Tukey’s test (*p* < 0.05) with data mean (n = 5). BS: Banana starch; ESP-BS: Eggshell powder–banana starch; OPP-BS: Orange peel powder–banana starch; ESP-OPP-BS: Eggshell–orange peel powder–banana starch.

**Table 4:** Comparison of removal percentage of biowaste biosorbents.

Biowaste Biosorbents	Target Analytes	Removal Percentage (%)	References
Eggshell powder	Pb(II)	48.21	[4]
Granular bentonite-eggshell composites	Pb(II)	99.90	[6]
Raw eggshell	Cu(II)	97.06	[7]
Orange peel	Cr(VI)	97.00	[10]
Modified orange peel	Cd(II)	98.00	[11]
Chitosan/orange peel hydrogel composite	Cr(VI), Cu(II)	80.43, 82.47	[13]
Orange peel	Cu(II), Pb(II)	88.00, 90.00	[34]
Orange peel	Cd(II)	88.34	[44]
Dragon fruit peel, rambutan peel, and passion fruit peel	Pb(II), Cd(II)	97.87, 97.10	[65]
Eggshell waste	Ni(II)	91.00	[66]
Kiwi, cucumber, and potato peel	Methylene blue	99.90	[67]
Eggshell and orange peel powder film	Cd(II)	99.95	This work

## Data Availability

Not applicable.

## References

[1] Food and Agriculture Organization (FAO) (2017). Strategic Work of FAO for Sustainable Food and Agriculture.

[2] Duque-Acevedo M., Belmonte-Urena L.J., Cortés-García F.J., Camacho-Ferre F. (2020). Agricultural waste: Review of the evolution, approaches and perspectives on alternative uses. Glob. Ecol. Conserv..

[3] Musonge P., Harripersadth C. (2021). The Applicability of Eggshell Waste as a Sustainable Biosorbent Medium in Wastewater Treatment—A Review. Adv. Wastewater Treat. I.

[4] Mahani N.A.A.M., Hamidon N. (2021). Eggshell Powder as an Adsorbent for Removal of Lead (II) in Panchorâ€™ s River. J. Adv. Manuf. Technol..

[5] Mittal A., Teotia M., Soni R.K., Mittal J. (2016). Applications of egg shell and egg shell membrane as adsorbents: A review. J. Mol. Liq..

[6] Wang G., Liu N., Zhang S., Zhu J., Xiao H., Ding C. (2022). Preparation and application of granular bentonite-eggshell composites for heavy metal removal. J. Porous Mater..

[7] Marković M., Gorgievski M., Štrbac N., Grekulović V., Božinović K., Zdravković M., Vuković M. (2023). Raw Eggshell as an Adsorbent for Copper Ions Biosorption—Equilibrium, Kinetic, Thermodynamic and Process Optimization Studies. Metals.

[8] Annane K., Lemlikchi W., Tingry S. (2021). Efficiency of eggshell as a low-cost adsorbent for removal of cadmium: Kinetic and isotherm studies. Biomass Convers. Biorefin..

[9] Xiao K., Liu H., Li Y., Yi L., Zhang X., Hu H., Yao H. (2018). Correlations between hydrochar properties and chemical constitution of orange peel waste during hydrothermal carbonization. Bioresour. Technol..

[10] Khalifa E.B., Rzig B., Chakroun R., Nouagui H., Hamrouni B. (2019). Application of response surface methodology for chromium removal by adsorption on low-cost biosorbent. Chemom. Intell. Lab. Syst..

[11] Tang H., Zhang Y., Zhang Y., Xiao Q., Zhao X., Yang S. (2022). Turning waste into adsorbent: Modification of discarded orange peel for highly efficient removal of Cd (II) from aqueous solution. Biochem. Eng. J..

[12] Altunkaynak Y., Canpolat M., Yavuz Ö. (2022). Adsorption of cobalt (II) ions from aqueous solution using orange peel waste: Equilibrium, kinetic and thermodynamic studies. J. Iran. Chem. Soc..

[13] Pavithra S., Thandapani G., Sugashini S., Sudha P.N., Alkhamis H.H., Alrefaei A.F., Almutairi M.H. (2021). Batch adsorption studies on surface tailored chitosan/orange peel hydrogel composite for the removal of Cr (VI) and Cu (II) ions from synthetic wastewater. Chemosphere.

[14] Lin Z., Chen L., Ye Z., Chen X., Wang X., Wei Y. (2021). Film-like chitin/polyethylenimine biosorbent for highly efficient removal of uranyl-carbonate compounds from water. J. Environ. Chem. Eng..

[15] Hu Y., Shi L., Ren Z., Hao G., Chen J., Weng W. (2021). Characterization of emulsion films prepared from soy protein isolate at different preheating temperatures. J. Food Eng..

[16] Vonnie J.M., Jing Ting B., Rovina K., Erna K.H., Felicia W.X.L., Nur ‘Aqilah N.M., Abdul Wahab R. (2022). Development of Aloe Vera-Green Banana Saba-Curcumin Composite Film for Colorimetric Detection of Ferrum (II). Polymers.

[17] Jridi M., Boughriba S., Abdelhedi O., Nciri H., Nasri R., Kchaou H., Kaya M., Sebai H., Zouari N., Nasri M. (2019). Investigation of physicochemical and antioxidant properties of gelatin edible film mixed with blood orange (*Citrus sinensis*) peel extract. Food Packag. Shelf Life.

[18] Akubor P.I., Igba T. (2019). Effect of pre gelatinization and annealing on the chemical composition, functional and pasting properties of starch prepared from unripe banana fruits. South Asian J. Food Technol. Environ..

[19] Rovina K., Vonnie J.M., Shaeera S.N., Yi S.X., Abd Halid N.F. (2020). Development of biodegradable hybrid polymer film for detection of formaldehyde in seafood products. Sens. Bio-Sens. Res..

[20] Sert D., Üçok G., Kara Ü., Mercan E.M.İ.N. (2021). Development of gelatine-based edible film by addition of whey powders with different demineralisation ratios: Physicochemical, thermal, mechanical and microstructural characteristics. Int. J. Dairy Technol..

[21] Dutta J., Devi N. (2021). Preparation, optimization, and characterization of chitosan-sepiolite nanocomposite films for wound healing. Int. J. Biol. Macromol..

[22] Khedri S., Sadeghi E., Rouhi M., Delshadian Z., Mortazavian A.M., de Toledo Guimarães J., Mohammadi R. (2021). Bioactive edible films: Development and characterization of gelatin edible films incorporated with casein phosphopeptides. LWT.

[23] Sree G.V., Nagaraaj P. (2022). Enhancement of PVA packaging properties using calcined eggshell waste as filler and nanonutrient. Mater. Chem. Phys..

[24] Cazón P., Vázquez M., Velazquez G. (2018). Novel composite films based on cellulose reinforced with chitosan and polyvinyl alcohol: Effect on mechanical properties and water vapour permeability. Polym. Test..

[25] Chávez-Salazar A., Bello-Pérez L.A., Agama-Acevedo E., Castellanos-Galeano F.J., Álvarez-Barreto C.I., Pacheco-Vargas G. (2017). Isolation and Partial Characterization of Starch from Banana Cultivars Grown in Colombia. Int. J. Biol. Macromol..

[26] Alias N.F., Ismail H. (2020). Glutaraldehyde Crosslinked Polyvinyl Alcohol/Eggshell Powder Biocomposite Films: Properties and Biodegradability. J. Phys. Sci..

[27] Durmus D. (2020). CIELAB color space boundaries under theoretical spectra and 99 test color samples. Color Res. Appl..

[28] Chareonsuk P., Mamaka N., Kulwongwit N., Wiriya-amornchai A., Bunroek P. (2021). The study of environmental stabilization for natural color dyed of eggshell powder filled in polylactic acid bio-composites. Mater. Today Proc..

[29] Kevij H.T., Salami M., Mohammadian M., Khodadadi M., Emam-Djomeh Z. (2021). Mechanical, physical, and bio-functional properties of biopolymer films based on gelatin as affected by enriching with orange peel powder. Polym. Bull..

[30] Terzioğlu P., Güney F., Parın F.N., Şen İ., Tuna S. (2021). Biowaste orange peel incorporated chitosan/polyvinyl alcohol composite films for food packaging applications. Food Packag. Shelf Life.

[31] Tibolla H., Pelissari F.M., Martins J.T., Lanzoni E.M., Vicente A.A., Menegalli F.C., Cunha R.L. (2019). Banana starch nanocomposite with cellulose nanofibers isolated from banana peel by enzymatic treatment: In vitro cytotoxicity assessment. Carbohydr. Polym..

[32] Wongphan P., Harnkarnsujarit N. (2020). Characterization of starch, agar and maltodextrin blends for controlled dissolution of edible films. Int. J. Biol. Macromol..

[33] Jiang B., Li S., Wu Y., Song J., Chen S., Li X., Sun H. (2018). Preparation and characterization of natural corn starch-based composite films reinforced by eggshell powder. CyTA-J. Food..

[34] Afolabi F.O., Musonge P., Bakare B.F. (2021). Application of the response surface methodology in the removal of Cu^2+^ and Pb^2+^ from aqueous solutions using orange peels. Sci. Afr..

[35] Yaradoddi J.S., Banapurmath N.R., Ganachari S.V., Soudagar M.E.M., Sajjan A.M., Kamat S., Mujtaba M.A., Shettar A.S., Anqi A.E., Safaei M.R. (2022). Bio-based material from fruit waste of orange peel for industrial applications. J. Mater. Res. Technol..

[36] Rápó E., Aradi L.E., Szabó Á., Posta K., Szép R., Tonk S. (2020). Adsorption of remazol brilliant violet-5R textile dye from aqueous solutions by using eggshell waste biosorbent. Sci. Rep..

[37] Sankaran R., Show P.L., Ooi C.W., Ling T.C., Shu-Jen C., Chen S.Y., Chang Y.K. (2020). Feasibility assessment of removal of heavy metals and soluble microbial products from aqueous solutions using eggshell wastes. Clean Technol. Environ. Policy.

[38] Pongsuwan C., Boonsuk P., Sermwittayawong D., Aiemcharoen P., Mayakun J., Kaewtatip K. (2022). Banana inflorescence waste fiber: An effective filler for starch-based bioplastics. Ind. Crops Prod..

[39] Kim D., Hwang S.J., Kim Y., Jeong C.H., Hong Y.P., Ryoo K.S. (2019). Removal of Heavy Metals from Water Using Chicken Egg Shell Powder as a Bio-Adsorbent. Bull. Korean Chem. Soc..

[40] Ji M., Li F., Li J., Li J., Zhang C., Sun K., Guo Z. (2021). Enhanced mechanical properties, water resistance, thermal stability, and biodegradation of the starch-sisal fibre composites with various fillers. Mater. Des..

[41] Dey S., Basha S.R., Babu G.V., Nagendra T. (2021). Characteristic and biosorption capacities of orange peels biosorbents for removal of ammonia and nitrate from contaminated water. Clean. Mater..

[42] Patel D.H., Naik J.H., Amaresan N. (2018). Synergistic effect of root-associated bacteria on plant growth and certain physiological parameters of banana plant (*Musa acuminata*). Arch. Agron. Soil Sci..

[43] Khoza M., Kayitesi E., Dlamini B.C. (2021). Physicochemical characteristics, microstructure and health promoting properties of green banana flour. Foods.

[44] Akinhanmi T.F., Ofudje E.A., Adeogun A.I., Aina P., Joseph I.M. (2020). Orange peel as low-cost adsorbent in the elimination of Cd (II) ion: Kinetics, isotherm, thermodynamic and optimization evaluations. Bioresour. Bioprocess..

[45] Iwase K., Mori K. (2020). Crystal structure, microhardness, and toughness of biomineral CaCO_3_. Cryst. Growth Des..

[46] Asri N.P., Podjojono B., Fujiani R. (2017). Utilization of eggshell waste as low-cost solid base catalyst for biodiesel production from used cooking oil. IOP Conference Series: Earth and Environmental Science.

[47] Adeogun A.I., Akande J.A., Idowu M.A., Kareem S.O. (2019). Magnetic tuned sorghum husk biosorbent for effective removal of cationic dyes from aqueous solution: Isotherm, kinetics, thermodynamics and optimization studies. Appl. Water Sci..

[48] Alimi B.A., Workneh T.S., Zubair B.A. (2022). Microstructural and physicochemical properties of biodegradable films developed from false banana (*Ensete ventricosum*) starch. Heliyon.

[49] Pelissari F.M., Andrade-Mahecha M.M., do Amaral Sobral P.J., Menegalli F.C. (2017). Nanocomposites based on banana starch reinforced with cellulose nanofibers isolated from banana peels. J. Colloid Interface Sci..

[50] Onwubu S.C., Vahed A., Singh S., Kanny K.M. (2017). Physicochemical characterization of a dental eggshell powder abrasive material. J. Appl. Biomater. Funct. Mater..

[51] Alimi B.A., Workneh T.S. (2018). Structural and physicochemical properties of heat moisture treated and citric acid modified acha and iburu starches. Food Hydrocoll..

[52] Vonnie J.M., Rovina K., Azhar R.A., Huda N., Erna K.H., Felicia W.X.L., Nur’Aqilah M.N., Halid N.F.A. (2022). Development and Characterization of the Biodegradable Film Derived from Eggshell and Cornstarch. J. Funct. Biomater..

[53] Fehlberg J., Lee C.L., Matuana L.M., Almenar E. (2020). Orange peel waste from juicing as raw material for plastic composites intended for use in food packaging. J. Appl. Polym. Sci..

[54] Chang Q., Li C., Sui J., Waterhouse G.I., Zhang Z.M., Yu L.M. (2022). Cage-like eggshell membrane-derived Co-CoxSy-Ni/N, S-codoped carbon composites for electromagnetic wave absorption. Chem. Eng. J..

[55] Özcan S., Çelebi H., Özcan Z. (2018). Removal of heavy metals from simulated water by using eggshell powder. Desalin. Water Treat..

[56] Wang R., Li X., Liu L., Chen W., Bai J., Ma F., Liu X., Kang W. (2020). Preparation and characterization of edible films composed of *Dioscorea opposita* Thunb. mucilage and starch. Polym. Test..

[57] Rasti A., Pineda M., Razavi M. (2020). Assessment of soil moisture content measurement methods: Conventional laboratory oven versus halogen moisture analyzer. J. Soil Water Conserv..

[58] Shafqat A., Al-Zaqri N., Tahir A., Alsalme A. (2021). Synthesis and characterization of starch based bioplatics using varying plant-based ingredients, plasticizers and natural fillers. Saudi J. Biol. Sci..

[59] Nogueira G.F., Fakhouri F.M., de Oliveira R.A. (2019). Effect of incorporation of blackberry particles on the physicochemical properties of edible films of arrowroot starch. Dry. Technol..

[60] Vonnie J.M., Li C.S., Erna K.H., Yin K.W., Felicia W.X.L., Aqilah M.N.N., Rovina K. (2022). Development of Eggshell-Based Orange Peel Activated Carbon Film for Synergetic Adsorption of Cadmium (II) Ion. Nanomaterials.

[61] Leites L.C., Frick P.J.M., Cristina T.I. (2021). Influence of the incorporation form of waste from the production of orange juice in the properties of cassava starch-based films. Food Hydrocoll..

[62] Chhatariya H.F., Srinivasan S., Choudhary P.M., Begum S.S. (2022). Corn starch biofilm reinforced with orange peel powder: Characterization of physicochemical and mechanical properties. Mater. Today Proc..

[63] Noshirvani N., Ghanbarzadeh B., Fasihi H., Almasi H. (2016). Starch–PVA nanocomposite film incorporated with cellulose nanocrystals and MMT: A comparative study. Int. J. Food Eng..

[64] Bhutto A.A., Baig J.A., Kazi T.G., Sierra-Alvarez R., Akhtar K., Perveen S., Afridi H.I., Ali H.E., Hol A., Samejo S. (2023). Biosynthesis of aluminium oxide nanobiocomposite and its application for the removal of toxic metals from drinking water. Ceram. Int..

[65] Wattanakornsiri A., Rattanawan P., Sanmueng T., Satchawan S., Jamnongkan T., Phuengphai P. (2022). Local fruit peel biosorbents for lead (II) and cadmium (II) ion removal from waste aqueous solution: A kinetic and equilibrium study. S. Afr. J. Chem. Eng..

[66] Angelis G.D., Medeghini L., Conte A.M., Mignardi S. (2017). Recycling of eggshell waste into low-cost adsorbent for Ni removal from wastewater. J. Clean. Prod..

[67] Mahmoodi N.M., Taghizadeh M., Taghizadeh A. (2018). Mesoporous activated carbons of low-cost agricultural bio-wastes with high adsorption capacity: Preparation and artificial neural network modeling of dye removal from single and multicomponent (binary and ternary) systems. J. Mol. Liq..

